# Oseltamivir Carboxylate, the Active Metabolite of Oseltamivir Phosphate (Tamiflu), Detected in Sewage Discharge and River Water in Japan

**DOI:** 10.1289/ehp.0900930

**Published:** 2009-09-28

**Authors:** Gopal C. Ghosh, Norihide Nakada, Naoyuki Yamashita, Hiroaki Tanaka

**Affiliations:** Research Centre for Environmental Quality Management, Graduate School of Engineering, Kyoto University, Japan

**Keywords:** influenza, LC-MS/MS, oseltamivir carboxylate, river water, sewage discharge, Tamiflu

## Abstract

**Background:**

Oseltamivir phosphate (OP; Tamiflu) is a prodrug of the anti-influenza neuraminidase inhibitor oseltamivir carboxylate (OC) and has been developed for the treatment and prevention of both A and B strains of influenza. The recent increase in OP resistance in influenza A virus (H1N1; commonlly called “swine flu”) has raised questions about the widespread use of Tamiflu in seasonal epidemics and the potential ecotoxicologic risk associated with its use in the event of a pandemic.

**Objectives:**

The objectives of this study were to develop an analytical method for quantitative determination of OC in sewage treatment plant (STP) effluent and receiving river water, and to investigate the occurrence of OC in STP effluent and river water in Japan during a seasonal flu outbreak.

**Methods:**

We developed an analytical method based on solid-phase extraction followed by liquid chromatography–tandem mass spectrometry. Using this method, we analyzed samples from three sampling campaigns conducted during the 2008–2009 flu season in Kyoto City, Japan.

**Results:**

The highest concentration of OC detected in STP discharge was 293.3 ng/L from a conventional activated-sludge–based STP; however, we detected only 37.9 ng/L from an advanced STP with ozonation as a tertiary treatment. In the receiving river water samples, we detected 6.6–190.2 ng/L OC, during the peak of the flu season.

**Conclusion:**

OC is present in STP effluent and river water only during the flu season. Ozonation as tertiary treatment in STP will substantially reduce the OC load in STP effluent during an influenza epidemic or pandemic.

Influenza virus infections continue to cause significant morbidity and mortality worldwide, and they place a considerable economic burden on individuals, families, businesses, and health care providers. Seasonal flu epidemics cause tens of millions of respiratory illnesses and 250,000–500,000 deaths worldwide each year [World Health Organization (WHO) 2003]. During the 20th century, flu pandemics caused millions of deaths, social disruption, and profound economic losses. The outbreak of the “Spanish flu” in 1918 was the worst, causing an estimated 40 million deaths worldwide, including 390,000 in Japan (Ministry of Health, Labour and Welfare of Japan 2005). Influenza pandemics occur when a new strain of the influenza virus is transmitted to humans from animals. Species thought to be important in the emergence of new human strains are ducks, chickens, and pigs. Recent emergences of highly pathogenic avian influenza virus (H5N1) and reports of flu virus resistance to antiviral drugs are of great concern. Two groups of antiviral drugs are used for the treatment of flu: the neuraminidase inhibitors (e.g., Tamiflu) and the M2 ion channel inhibitors (e.g., amantadine). Oseltamivir phosphate (OP; Figure 1), marketed as Tamiflu, is recommended by the WHO for both treatment of influenza and prophylaxis and is considered an important first-line defense in the event of a flu pandemic. OP is a prodrug that is rapidly and extensively hydrolyzed *in vivo* to its active metabolite, oseltamivir carboxylate (OC; Figure 1), a potent and selective inhibitor of influenza A and B virus neuraminidase. OC is excreted (> 80% of oral dose) unchanged (Sweetman 2007).

OP is widely used in Japan. As with many other pharmacologically active compounds, sewage treatment plant (STP) effluent is the main source of OC in the environment because OC is not removed significantly in STPs (Fick et al. 2007). The most widespread subtypes of influenza A virus are transmitted through waterfowl, where they remain, multiply, and are excreted in large quantities in droppings (Olsen et al. 2006). Notably, waterfowl stay close to STP discharge points, where the water temperature is higher and where they find adequate food, especially in winter, the flu season. Therefore, widespread use of OP to fight seasonal influenza in humans could lead to the development of OC-resistant strains of the viruses in wild birds (Fick et al. 2007; Singer et al. 2007). In addition, a mass administration of OP during a future pandemic could pose a risk to drinking water safety and ecologic health.

In the present study we developed a method for the detection of OC in STP discharge and in river water using solid-phase extraction (SPE) followed by liquid chromatography–tandem mass spectrometry (LC-MS/MS), and investigated the occurrence of OC in STP effluent and receiving river water during the 2008–2009 flu season in Kyoto City, Japan.

## Materials and Methods

### Chemicals and reagents

OC and deuterium-labeled OC (OC-D_3_) standards were provided by F. Hoffmann-La Roche Ltd. (Pharma Research, Basel, Switzerland). Acetonitrile (LC-MS grade), methanol (LC-MS grade), formic acid, ascorbic acid, sodium chloride, and ammonia solution (25%) were purchased from Wako Pure Chemical Industry, Ltd. (Osaka, Japan). Individual standard stock solutions of OC and OC-D_3_ (1 mg/10 mL), prepared by weighing and dissolving the compounds in methanol, were stored at − 20°C. All working standards were prepared from stock solution within 2 days before analysis and stored at 4°C for a maximum of 3 days.

### Site description

**Figure 2:**
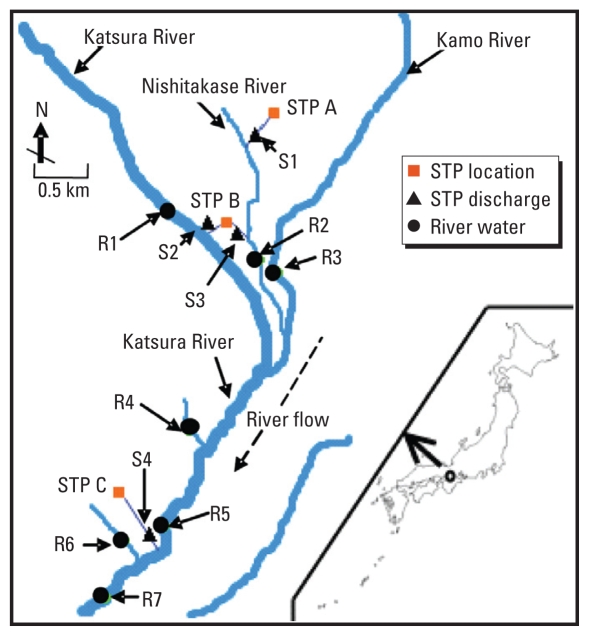
Location of sampling points in the context of Japan (inset). S1 (STP A), S2 (STP B), S3 (STP B), and S4 (STP C) are sampling points for STP discharges. R1–R7 indicate sampling points for river water.

All sampling points were located in the Katsura River catchment, covering river water and STP discharges. The Katsura River receives high anthropogenic inputs, including > 80% of wastewater generated from Kyoto City (1,389,000 people) and is the final outlet of STPs A, B, and C (Figure 2). STP B has two discharge points, one to the Katsura River (S2) and the other to the Nishitakase River (S3; Figure 2); around 90% of the water in the Nishitakase River during dry weather comes from STPs A and B. River-water sampling point R1 was the most upstream point on the Katsura River, and R7 was the most downstream point.

### Extraction method optimization

We adjusted the pH of Milli-Q water (Millipore, Billerica, MA) with 0.1 M sulfuric acid or 10% ammonia solution to 4.0, 7.0, and 11.0 to examine the effect of pH on the extraction efficiency of the SPE cartridge. To study breakthrough, we tested volumes of 100, 300, 500, and 1,000 mL using OC-free samples spiked with OC and OC-D_3_ at 50 ng/L, and we enriched sample matrixes on two stacked cartridges.

### Sample collection and preparation

We assayed for OC in STP effluent and receiving river water during three 1-day-long sampling campaigns (dry weather conditions): the weeks beginning 4 December 2008, 4 February 2009, and 24 February 2009. Samples were collected from 11 locations: sampling points S1–S4 refer to STP discharge sites, and R1–R7 are to river sites (Figure 2). Sewage effluent samples were always gathered from the outlet of STPs, and river samples from a bridge over the center of the stream. No samples were collected at R2 during the first and third campaigns or from S4 during the third campaign.

Grab samples (300 mL) were collected in glass bottles containing ascorbic acid (1 g/L) to acidify the samples and for chlorine and ozone quenching. All the samples were filtered (Whatman GF/B glass microfiber filter, 1 μm pore size) as soon as possible, but no more than 5 hr after sampling. The filtered samples were immediately extracted or kept at − 25°C in half-filled amber glass bottles laid horizontally until extraction. Before extraction, sodium chloride (1 g/L) was added and the pH was adjusted to 4.0 with sulfuric acid to enhance OC extraction yield by SPE; the surrogate standard OC-D_3_ (50 ng) was added to calculate the recovery rate. SPE was performed with 6-cc Oasis HLB sorbent cartridges (200 mg; Waters Co., Milford, MA, USA) using a Chratec Sep-Pak Concentrator (SPC 10-C; Chratec, Kyoto, Japan). The cartridges were preconditioned with 3 mL methanol followed by 3 mL Milli-Q water (pH 4.0). All samples were passed through the cartridges at a flow rate of 5 mL/min. After concentration, the cartridges were dried completely with air on a vacuum manifold for 2 hr. The analytes were then eluted from the cartridge with 6 mL methanol containing 2% ammonia, passed through a Sep-Pak Plus NH2 (360 mg; Waters) cartridge for cleanup into 10-mL graduated glass vessels, and dried by a gentle flow of nitrogen at 37°C. The final sample volume was adjusted to 0.5 mL with 0.1% formic acid in Milli-Q water containing 20% acetonitrile (a concentration factor of 600).

### LC-MS/MS

OC and OC-D_3_ were separated in a Waters Acquity Ultra Performance LC (UPLC) separation module with a binary pump system equipped with a UPLC BEH C18 column (100 mm × 2.1 mm, 1.7 μm particle size). Optimum separation was achieved with a binary gradient of 0.1% formic acid (vol/vol) in water (solvent A) and acetonitrile (solvent B) at a flow rate of 0.35 mL/min under the following program: 0–2 min, 10% B; 2–4 min, 10–75% B; 4–4.3 min, 75–90% B; 4.3–5.3 min, 90% B; 5.3–5.8 min, 90–10% B; 5.8–8 min, 10% B (equilibration of column). The column temperature was kept at 60°C, and the injected sample volume was 10 μL. The UPLC system was coupled to a Quattro Micro API MS with electrospray ionization (Waters).

During quantification optimization, OC and OC-D_3_ were individually infused as standard solutions into the initial mobile phase (50% solvent A + 50% solvent B) directly into the MS at 1 mg/L at a cone voltage of 20 V, a collision energy of 10 eV, a product ion *m*/*z* of 285.06 (OC) or 288.07 (OC-D_3_), a daughter ion *m*/*z* of 196.72 (OC) or 199.71 (OC-D_3_), and a retention time of 3.24 min. The MS used an electrospray source block temperature of 120°C and a desolvation temperature of 400°C, a capillary voltage of 2.5 kV, and cone and desolvation gas flows of 50 and 900 L/hr, respectively. Instrument control, data acquisition, and quantification were performed by Mass Lynx 4.1 software (Waters).

### Method validation

We anlyzed OC and OC-D_3_ by multiple reaction monitoring at the highest precursor and product ion transitions. Positive identification of OC was based on LC retention time compared with that of OC-D_3_ surrogate standard, and < 0.05% deviation of retention time was tolerated. Seven-point calibration curves were constructed for quantification, ranging between 0.5 and 400 ng/mL. The accuracy of the overall analytical procedure was evaluated with blank river water and STP discharge samples, that is, free of OC (collected on 12, 17, and 30 June 2008 during dry weather in the non-flu season), spiked at 100 ng/L and analyzed in quintuplicate, and compared with a direct injection of a standard mixture at the same concentration. Blank samples were previously analyzed to confirm the absence of any significant peak at the selected transition. Reproducibility was assessed by injection of OC-free sewage discharge and river water spiked at 50 ng/L (*n* = 5). The method was considered accurate if recoveries were in the range of 70–140%, and precision was satisfactory if the relative SD was < 15%. The instrumental limit of detection (LOD) and limit of quantification (LOQ) were calculated from the SD of the repeated measurement (*n* = 5) of a standard solution (150 pg on column): LOD = 3 SD; LOQ = 10 SD. In a sample, OC was considered to be quantifiable when the signal of the peak was ≥ 10× the background noise.

### Prediction of OC in STP discharge

The concentration of OC in STP discharge on a given day was calculated using the following equation:


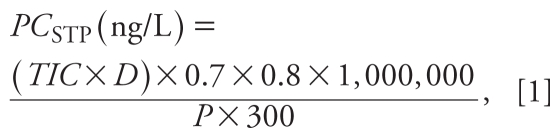


where *PC*_STP_ is the predicted concentration of OC in STP discharge in Kyoto City; *TIC* is the total number of influenza cases per week in Kyoto City during the study period; *D* is the OP dose assuming 85% adults (2 × 75 mg OP/day) and 15% children (2 × 45 mg OP/day), reflecting the population structure of Kyoto City in 2008; 0.7 reflects that on any day in the week only five of seven patients will be taking OP because the treatment course is 5 days; 0.8 represents the 80% of ingested OP that is excreted unaltered; *P* is the total population of Kyoto City (1,389,000); and 300 is the average per capita water use (liters per person per day). Further, we assumed that all confirmed influenza cases were treated with OP and that there was zero degradation of OC in the conventional STPs (B and C) (Fick et al. 2007). This assumption is not applicable to STP A, which uses ozonation-based tertiary treatment, which efficiently removes pharmaceuticals in wastewater and drinking water (Huber et al. 2003; Ternes et al. 2003).

## Results and Discussion

### Method validation and application

The pH of the sample proved to be the most influential variable during sample enrichment on the SPE cartridge. Extraction efficiency decreased as pH increased: it was 22% less at pH 11.0 than at pH 4.0. We therefore selected pH 4.0. We used a sample volume of 300 mL, which was the maximum volume without a cartridge breakthrough.

**Figure 3:**
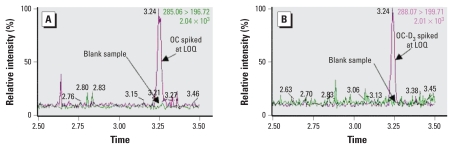
Representative total ion chromatograms of (*A*) a blank sewage discharge sample (OC-free) and spiked OC at the LOQ and (*B*) a blank sewage discharge sample and spiked OC-D_3_ at the LOQ of OC.

**Figure 4:**
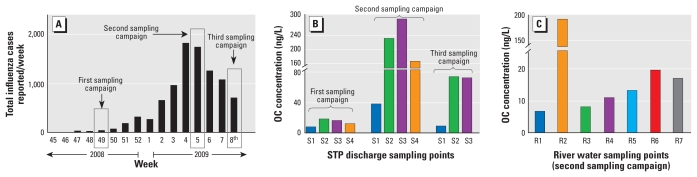
(*A*) Total number of influenza cases reported per week during the 2008–2009 influenza outbreak in Kyoto City during three sampling campaigns. (*B*) OC concentrations at STPs during three sampling campaigns (no sample was collected at S4 during the third campaign). (*C*) OC concentrations in river water during the second campaign. OC was detected in river water only during the second sampling campaign; no sample was collected at R2 during the first and third campaigns.

The extraction yield of the surrogate standard OC-D_3_ was analogous to that of OC, as expected, owing to their similarities in physicochemical properties. Thus, the analyte was quantitatively enriched by one cartridge and exhaustively eluted as described in “Materials and Methods.” The Sep-Pak Plus NH2 cartridge for cleanup during elution reduced matrix effects and markedly increased the peak sharpness and instrument sensitivity. The standard curves were linear, with correlation factors typically > 0.99, between 5 and 4,000 pg on the column. The precision of the entire method (reproducibility) ranged between 2% and 7.4%. The resulting recoveries were 128% ± 11% from STP effluent and 110% ± 8% from river water. The extraction yield in the sample was determined from peak area ratios of analyte and surrogate standard. The method LOD and LOQ for OC were 3.6 and 12 ng/L, respectively. Representative chromatograms of OC and OC-D_3_ at LOQ are shown in Figure 3. The method was successfully applied to the analyses of river water and STP samples (Figure 4).

### OC in STP discharge and river water

We measured OC concentration only in liquid phase (GF/B filtered sample). OC has both amine and carboxylate groups, a low partition coefficient (log *P* of 1.1), and a high water solubility (588 mg/mL at 25°C) (Singer et al. 2007). These physicochemical properties indicate a very low potential for sorption to suspended solids during treatment, so the load in the solid phase in sewage effluent will be low. We found OC in all STP discharges during all three sampling campaigns, at concentrations up to 293.3 ng/L (Figure 4B).

**Figure 5:**
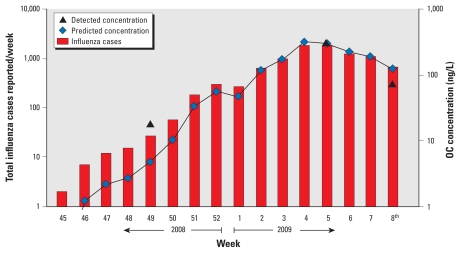
Total number of influenza cases reported per week with predicted and maximum detected concentrations of OC in Kyoto City sewage discharges (see Equation 1 for assumptions).

The first sampling campaign ran at the beginning of the flu season (Figure 4A). Only 27 confirmed cases were reported that week; OC was detected in all STP discharges but not in the river water samples (Figure 4B). The highest concentration (18.2 ng/L) was detected at sampling point S2, a discharge point of STP B, an activated-sludge–based STP (Table 1). A slightly lower concentration (16.2 ng/L) was detected at point S3, another discharge point of STP B. During sampling, the average flow rates were 431,940 m^3^/day at S2 and 105,150 m^3^/day at S3, implying an OC load of 7.8 g/day to the Katsura River and 1.7 g/day to the Nishitakase River. STP C, another activated-sludge–based STP (Table 1), also discharges to the Katsura River. At S4, the OC concentration was 12.0 ng/L, with an OC load of 1.6 g/day. The predicted OC concentration (Equation 1) in STP discharge was 4.0 ng/L, much lower than the maximum concentration detected at STP B (Figure 5). Our prediction was based on confirmed flu cases reported by the Kyoto City office, so there could be a gap between the doses of Tamiflu taken and the number of confirmed cases. The results also suggest the preventive use of Tamiflu (75 mg/day for 10 days) to help control flu outbreaks among people at high risk, such as in nursing homes or hospital wards. This would explain our underestimate. The OC concentration was 6.3 ng/L at point S1 (STP A), with an OC load of 0.35 g/day to the Nishitakase River, substantially lower than at STPs B and C (this was true of all campaigns). STP A uses ozonation (4 mg ozone/L for an average contact time of 10 min) as a tertiary treatment (Table 1). This is the key factor in the lower level of OC at STP A. This finding reveals that the assumption of zero removal of OC may not be appropriate for the prediction of concentration in effluent without an evaluation of the effect of ozonation on OC.

The second sampling campaign ran during the peak period of the 2008–2009 flu season in Kyoto City. The city recorded 1,738 cases during week. OC was detected at all sampling points, with the highest concentration at point S3 (293.3 ng/L). The predicted concentration of OC in STP discharge was 311 ng/L, very close to the actual maximum. The number of patients increased by a factor of 60 between the first and second campaigns, but the concentration of OC in STP discharge increased by a factor of only 20. This difference could be due to a decrease in the prophylactic use of Tamiflu since the beginning of the flu outbreak and to the use of Zanamivir (another neuraminidase inhibitor; GlaxoSmithKline, Tokyo, Japan) instead of Tamiflu in some cases, because Tamiflu-resistant H1N1 virus was recorded during the 2008–2009 flu season in Japan (WHO 2009). At sampling point R1, in the Katsura River upstream of STP B (S2), the river incorporates wastewater from a small city and hilly areas, and had a relatively low concentration of OC (6.6 ng/L) (Figure 4C). Similarly, the concentration was low at Kamo River sampling point R3 (8.2 ng/L), which also has low levels of anthropogenic contaminants. On the other hand, the Nishitakase River carries treated sewage from STP A (S1) and STP B (S3), accounting for around 90% of its total flow, and had a high concentration of OC at R2 (190.2 ng/L), as expected. The Nishitakase and Kamo rivers join the Katsura River upstream of R5, where the OC concentration was 13.1 ng/L, comparable to that at the most downstream point, R7 (11.6 ng/L). The OC concentrations at R4 and R6 were 11.3 and 19.6 ng/L, respectively.

During the third sampling campaign, OC was again detected only in STP effluent: 9.0 ng/L at S1, 74.6 ng/L at S2, and 72.7 ng/L at S3.

### Transport, dissipation, and degradation

During the non-flu period (June–October 2008), OC was not detected in sewage discharge (S1, S2, S3) or river water (R5). The Katsura River is the main river in the study area, receiving most of the wastewater generated from Kyoto City. The OC concentration increased from point R1 (6.6 ng/L) downstream to R5 (13.1 ng/L) during the flu season (Figure 4C). Discharges from STPs A and B were the main sources involved in this increase. There was no effect of dilution between R1 and R5 because a higher concentration of OC was detected at the Kamo River sampling site (R3; 8.2 ng/L) than at R1. The OC concentration at the most downstream point on the Katsura River (R7; 17.1 ng/L) was higher than that at R5. The addition of OC from STP C effluent (S4; 172.0 ng/L) and a small canal between R5 and R7 (R6; 19.6 ng/L) was responsible for the higher concentration at R7 than at R5. As is the case for other pharmaceuticals, OC may degrade by sorption, biodegradation, and photolysis. In river water, OC can be degraded by the addition of a small amount (5%) of sediment, which promotes microbial degradation but requires several weeks (> 8% of ^14^C-OC evolved as ^14^CO_2_ from water/sediment samples after 21 days of incubation) (Saccà et al. 2009). Because OC has a low sorption affinity for sediment, sorption would explain little degradation (Saccà et al. 2009). Because the river flow in the study area is fast (complete transit in a few hours), the removal of OC by sorption and biodegradation between R1 and R7 will be insignificant. On the other hand, direct photolysis plays little or no role in the decomposition of OC: The half-life of OC in unfiltered Elbe River water was 427 hr (Bartels and Tümpling 2008). Thus, OC will persist in water, and a substantial load reduction of OC from STPs may be required in the event of an influenza epidemic or pandemic.

STPs A, B, and C are located in Kyoto City and treat mainly domestic wastewater. STP A also receives industrial effluent from small-scale factories that manufacture kimonos (traditional Japanese dress), accounting for < 10% of its total volume. The samples were collected during dry weather, and surface runoff was negligible. Under these circumstances, the OC concentration in the STP A discharge should be similar to those at STPs B and C. Yet during the second sampling campaign, the maximum OC concentrations were 37.9 ng/L at STP A, 293.3 ng/L at STP B, and 172 ng/L at STP C. Ozonation at STP A explains the low concentration there (Huber et al. 2003; Ternes et al. 2003). We conclude that ozonation will substantially reduce the OC load in STP effluent during an influenza epidemic or pandemic.

### Environmental risk

The highest OC concentrations were detected during the second sampling campaign: 293.3 ng/L at STP A site S3 and 190.2 ng/L at Nishitakase River site R2. The Nishitakase carries mostly STP A and STP B effluent on dry days, resulting in its high concentration. The predicted environmental levels of OC during a flu pandemic in Europe and North America ranged from < 300 ng/L to 32 μg/L, depending on the characteristics of the river basins (Singer et al. 2007). In the Katsura River, the highest concentration was 17.1 ng/L at R7, the most downstream point in the present study. In an acute toxicity study using *Daphnia magna*, Tamiflu was classified harmful according to European Union Directive 67/548/EEC as amended (European Medicines Agency 2005). However, on the basis of “predicted environmental concentration/predicted no-effect concentration” risk ratios, no significant risk of surface waters or STP effluent during both seasonal and pandemic use of Tamiflu was evident (Straub 2009). Nitrification in STPs is generally considered to be sensitive to toxic compounds (Pagga et al. 2006). Yet concentrations of OC up to 20 μg/mL had no observed impact on the structure of the microbial community or on bacterial nitrification processes (Saccà et al. 2009). The risk of OC resistance in wildfowl was considered less important than the synergistic effects of pharmaceuticals in sewage on the biological sewage treatment process (Singer et al. 2008). However, the concentration that inhibited 50% of influenza virus (IC_50_) ranged between 80 and 230 ng/L (Gubareva et al. 2001; Monto et al. 2006), similar to the range detected in STP discharge here. The emergence of resistance to Tamiflu in the influenza virus H1N1 during the 2008–2009 flu season are of great concern (WHO 2009). During a common flu season, waterfowl can ingest large quantities of OC with virus (virus is believed to be transmitted among waterfowl by the fecal–oral route) in their daily water intake, and a high percentage of OC ingested by waterfowl will remain in the intestinal tract, the primary site of viral replication, possibly promoting drug resistance (Fick et al. 2007; Singer et al. 2007).

## Conclusion

We developed and validated an LC-MS/MS–based analytical method for the determination of OC in water samples. Here, we report for the first time the detection of OC in STP discharges and river water during a flu season. The highest concentration of OC detected in STP discharge was 293.3 ng/L at an STP operating with only an activated-sludge–based treatment system. One STP operating with ozonation as a tertiary treatment was highly efficient at removing OC (> 85% from secondary effluent). Ozonation as tertiary treatment in STP will substantially reduce the OC load in STP effluent during an influenza epidemic or pandemic. Further research should investigate the fate of antiviral drugs during every process at every unit in the STPs.

## Figures and Tables

**Figure 1:**
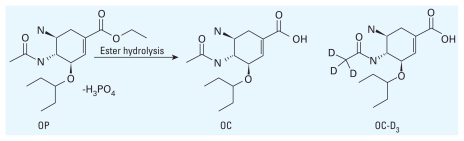
Structure of the prodrug OP, its active metabolite OC, and OC-D_3_.

**Table 1:** STP characteristics.

			Treatment processes		
STP	Capacity (m^3^/day)	Population served	Primary	Secondary	Tertiary
A	114,000	83,000	Settling	AOAO	O_3_
B	975,000	773,000	Settling	CAS, A2O, AOAO	
C	228,000	338,167	Settling	AOAO	

Abbreviations: A2O, anaerobic/anoxic/oxic; AOAO, anoxic/oxic/anoxic/oxic; CAS, conventional activated sludge; O_3_^,^ ozonation (4 mg ozone/L; average contact time, 10 min).
